# Towards Novel HIV-1 Serodiagnostic Tests without Vaccine-Induced Seroreactivity

**DOI:** 10.1128/spectrum.00715-23

**Published:** 2023-05-24

**Authors:** Ole Lagatie, Dax Lauwers, Harvinder Singh, Fien Vanroye, Daniel J. Stieh, Johan Vingerhoets, Ludo Lavreys, Valérie Oriol-Mathieu, Will Colón, Chris Verhofstede, Koen Vercauteren, Dorien Van den Bossche, Maria Grazia Pau

**Affiliations:** a Johnson & Johnson Global Public Health Research & Development, Beerse, Belgium; b Institute of Tropical Medicine, Antwerp, Belgium; c Janssen Vaccines and Prevention B.V., Leiden, The Netherlands; d Janssen Research & Development, Beerse, Belgium; e Ghent University, Ghent, Belgium; Kumamoto Daigaku

**Keywords:** antigens, diagnostics, human immunodeficiency virus, seroreactivity, vaccine

## Abstract

Vaccine-induced seroreactivity/positivity (VISR/P) poses a significant and common challenge to HIV vaccine implementation, as up to 95% of vaccine recipients may be misclassified as having HIV infection by current HIV screening and confirmatory serological assays. We investigated whether internal HIV proteins could be used to overcome VISR and discovered a set of 4 antigens (gp41 endodomain, p31 integrase, p17 matrix protein, and Nef) that are recognized by antibodies produced in individuals with HIV infection but not in vaccinated individuals. When evaluated in a multiplex double-antigen bridging ELISA, this antigen combination had specificities of 98.1% prevaccination and 97.1% postvaccination, demonstrating the assay is minimally impacted by vaccine-induced antibodies. The sensitivity was 98.5%, further increasing to 99.7% when p24 antigen testing was included. Results were similar across HIV-1 clades. Although more technical advancements will be desired, this research provides the groundwork for the development of new fourth-generation HIV tests unaffected by VISR.

**IMPORTANCE** While the detection of HIV infection is accomplished by several methods, the most common are serological tests that detect host antibodies produced in response to viral infection. However, the use of current serological tests may present a significant challenge to the adoption of an HIV vaccine in the future because the antibodies to HIV antigens detected in currently available tests also tend to be included as antigens in the HIV vaccines in development. The use of these serological tests may thus result in the misclassification of vaccinated HIV-negative individuals, which can have potential for significant harms for individuals and could prevent the widespread adoption and implementation of HIV vaccines. Our study aimed to identify and evaluate target antigens for inclusion in new serological tests that can be used to identify HIV infections without interference from vaccine-induced antibodies but also fit within existing platforms for HIV diagnostics.

## INTRODUCTION

With 1.5 million new infections and approximately 680,000 lives lost to AIDS-related illnesses in 2020, HIV remains a major world health problem (1). While the detection of HIV infection is accomplished by several methods, including the identification of viral proteins and the detection of viral nucleic acids in the blood of individuals living with HIV, the most common method detects antibodies produced in response to viral infection rather than the virus itself (2–5). HIV serological tests are accessible, inexpensive, and easy to use, which contributes to their widespread use (6).

Although a vaccine to protect against HIV infection is greatly needed, the use of current serological tests may present a significant challenge to the adoption of an HIV vaccine in the future. The goal of vaccination is to create an antibody response to HIV viral antigens, which may result in the misclassification of vaccinated HIV-1–negative individuals due to vaccine-induced seroreactivity/positivity (VISR/P) (7, 8). Antibodies to HIV antigens detected in currently available serology-based screening and confirmatory tests, such as the envelope (Env) glycoproteins gp120 and gp41, also tend to be included as antigens in the HIV vaccines in development (9, 10). A majority of individuals who have received a mosaic-based HIV-1 vaccine regimen developed by Janssen in clinical trials have experienced VISR (7, 9, 11). This mosaic-based vaccine consists of a combination of adenovirus type 26 (Ad26) vectors expressing the globally relevant group M bivalent mosaic Env/Gag/Pol antigens and a protein-based vaccine component that contains trimeric HIV gp140 soluble proteins. The incidence of VISR reported in HIV vaccine studies has ranged widely from 0.4% to >95% across vaccine developers, depending on the type of serological test used, participant characteristics, and vaccine design, potency, durability, and dosing (7, 9, 11–13). The duration of VISR also varies, with antibodies lasting >20 years in some cases (14). In addition, the transmission of VISR may occur through blood and organ donation, as well as from mother to child (7).

Incorrectly classifying an uninfected vaccinated individual as being infected with HIV has the potential for significant harm. Individuals with VISR may experience social harms, such as misunderstandings with family, friends, and health care workers; may be unable to donate blood or organs; and may face challenges with insurance, military service, employment, travel, immigration, and pregnancy (7). Misclassification due to VISR may also result in unnecessary medical follow-up and/or treatment. Further, VISR may prevent the identification of breakthrough HIV infections in vaccinated individuals in cases where a positive serological test is incorrectly attributed to VISR and not to a true HIV infection (7), leading to a delay in treatment or no treatment at all and risk of transmission, which are also of significant concern.

When VISR is present, only viral nucleic acid tests can currently detect an actual HIV infection; however, these tests are associated with cost and logistical barriers in many regions of the world, which may complicate vaccine adoption, especially in resource-limited settings (7, 9). Current serological screening tests detect antibodies against several HIV antigens and, in most cases, also detect the p24 antigen itself to ensure high specificity and sensitivity; while these tests will not be viable for HIV diagnosis following the adoption of future HIV vaccines, the most efficient strategy would be to adapt these existing testing platforms by changing the HIV antigens they detect antibodies against. Prior attempts to design serological tests capable of discriminating between vaccine- and infection-induced seroreactivity have focused on the use of a set of specific HIV-1 peptides (15–18). Unfortunately, the development of a diagnostic test based on these peptides has been discontinued.

The aim of this study was to identify and evaluate target antigens for inclusion in new serological screening tests that can be used to identify HIV infections without interference from vaccine-induced antibodies but also fit within existing platforms for HIV diagnostics. We named these updated serological screening tests DHIVAx, for Diagnosis of HIV in a Vaccinated world. Ideally, DHIVAx tests should include a set of HIV antigens with the following characteristics: (i) internal proteins less likely to be exposed to the immune system in the context of an HIV vaccine, (ii) highly immunogenic in individuals infected with HIV, (iii) not recognized by HIV vaccine–induced antibodies, (iv) recognized by antibodies from individuals infected with HIV early postinfection (i.e., as soon as p24 antigen becomes undetectable), (v) maintaining detection of antibody response over all stages of the disease, and (vi) enabling detection of antibodies against all HIV clades and subtypes. Our approach would require changing only key ingredients of current tests (i.e., the HIV antigens) without changing the test format, instruments, assay procedures, and interpretation of results.

## RESULTS

### Identification of HIV-1 candidate antigens.

**FIG 1:**
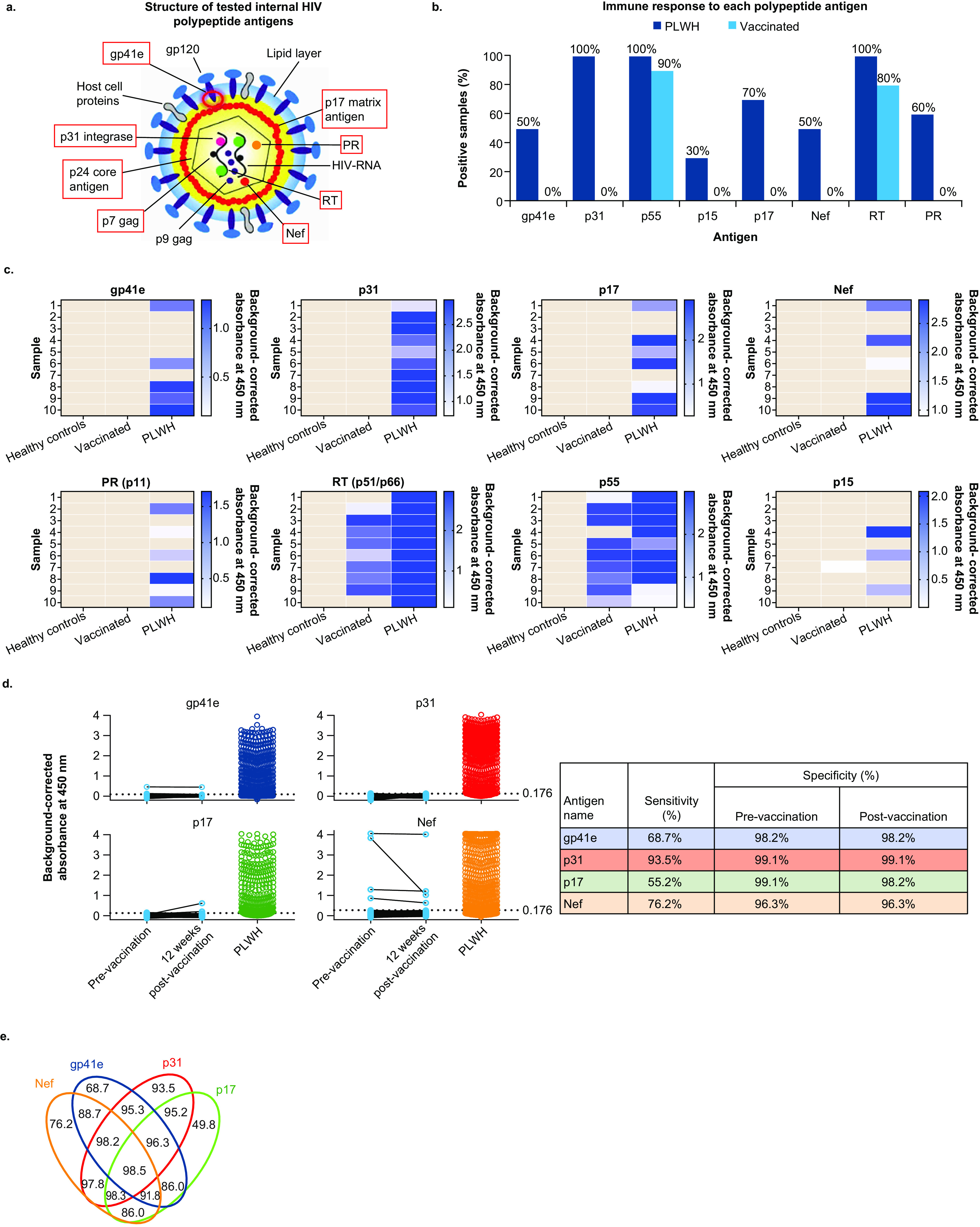
Evaluation and performance of individual HIV-1 candidate antigens. (a) Structure of HIV indicating polypeptide antigens that were tested. (b) Immune response to each polypeptide antigen in individuals living with known HIV-1 infection and those who participated in HIV vaccine studies. (c) Heat maps of serological assays for the identification of antigens. Samples shaded gray were below the cutoff 0.1 μg/mL for gp41e and PR, 0.7 μg/mL for p31, 0.25 μg/mL for p17, 0.9 μg/mL for Nef, 0.1 U/mL for RT, 0.3 μg/mL for p55, and 0 μg/mL for p15. (d) Performance evaluation of individual HIV-1 candidate antigens. Sensitivity of each polypeptide antigen to detect infection in treatment-naive individuals living with known HIV-1 infection (AMBER, *n* = 600). The specificity of each antigen was evaluated in individuals who participated in HIV vaccine studies (TRAVERSE and ASCENT, *n* = 109). Dotted horizontal lines represent the threshold for positivity (0.176). (e) Venn diagram sensitivities for different combinations of indirect ELISA. gp41e, glycoprotein 41 endodomain; PLWH, people living with HIV; PR, protease; RT, reverse transcriptase.

A number of HIV-1 antigens, mainly internal, were selected as diagnostic candidates, as these internal viral proteins are less likely to be exposed to the immune system in the context of an HIV vaccine, based on current knowledge. Of the initial set of 8 HIV-1 candidate polypeptide antigens evaluated, 5 (gp41 endodomain [gp41e], integrase p31 [p31], matrix antigen p17 [p17], protease [PR], and Nef) were found to elicit little or no immunoreactivity in individuals (*n* = 10) vaccinated with the mosaic-based HIV-1 vaccine regimen (from the TRAVERSE and ASCENT studies [19, 20]) but were reactive in at least half of individuals (*n* = 10) with known HIV-1 infection (Fig. 1A to C). Of note, reverse transcriptase (RT) and p55 were also found to be strong markers for the detection of HIV infection; however, they were not selected for further evaluation as candidate diagnostic antigens because of the high reactivity against these antigens after receiving the vaccines used in these studies.

### Performance evaluation of individual HIV-1 candidate antigens.

Four of the 5 identified polypeptide antigens (gp41e, p31, p17, and Nef) were selected for optimization of an indirect enzyme-linked immunoassay (ELISA; Fig. 1D) to lower the nonspecific binding of antibodies in uninfected individuals. PR was not included, as the proteolytic function of this protein might complicate the downstream application of this antigen. The optimized assays were used to determine the immunoreactivity in a set of serum/plasma samples from 600 treatment-naive individuals with known HIV-1 infection enrolled in the AMBER study (21) and 109 healthy uninfected participants of the TRAVERSE and ASCENT studies evaluating the mosaic vaccine (19, 20). The individual polypeptide antigens had sensitivities to detect HIV infection of 68.7%, 93.5%, 55.2%, and 76.2% for gp41e, p31, p17, and Nef, respectively.

The specificity in healthy uninfected individuals both before and after full vaccination was >98.0% for all antigens, except for Nef, which had a specificity of >96.0% (Fig. 1D). Of the 109 healthy uninfected individuals, 7 (6.4%) were positive for ≥1 of the polypeptide antigens before vaccination and 8 (7.3%) were positive after vaccination (including 4 individuals who were positive before vaccination), indicating no VISR.

Although no individual polypeptide antigen provided adequate sensitivity and specificity, they do show complementarity in their reactivity with sera from individuals living with HIV. As a result, the individual sensitivities combined for an overall sensitivity of 98.5% (Fig. 1E), which increased to 99.0% when p24 antigen detection was also included. The antigens were similarly recognized by samples from HIV-infected and seropositive individuals from different geographical origins and who were infected with different HIV-1 clades (Table S1). Most samples that were negative for all 4 antigens were from HIV-infected individuals who were recently diagnosed (<12 weeks since the first diagnosis; Table S2). The sensitivity for samples >12 weeks since the first diagnosis was 100.0%.

### Performance evaluation of the combined HIV-1 candidate antigens in a double-antigen bridging ELISA.

**FIG 2:**
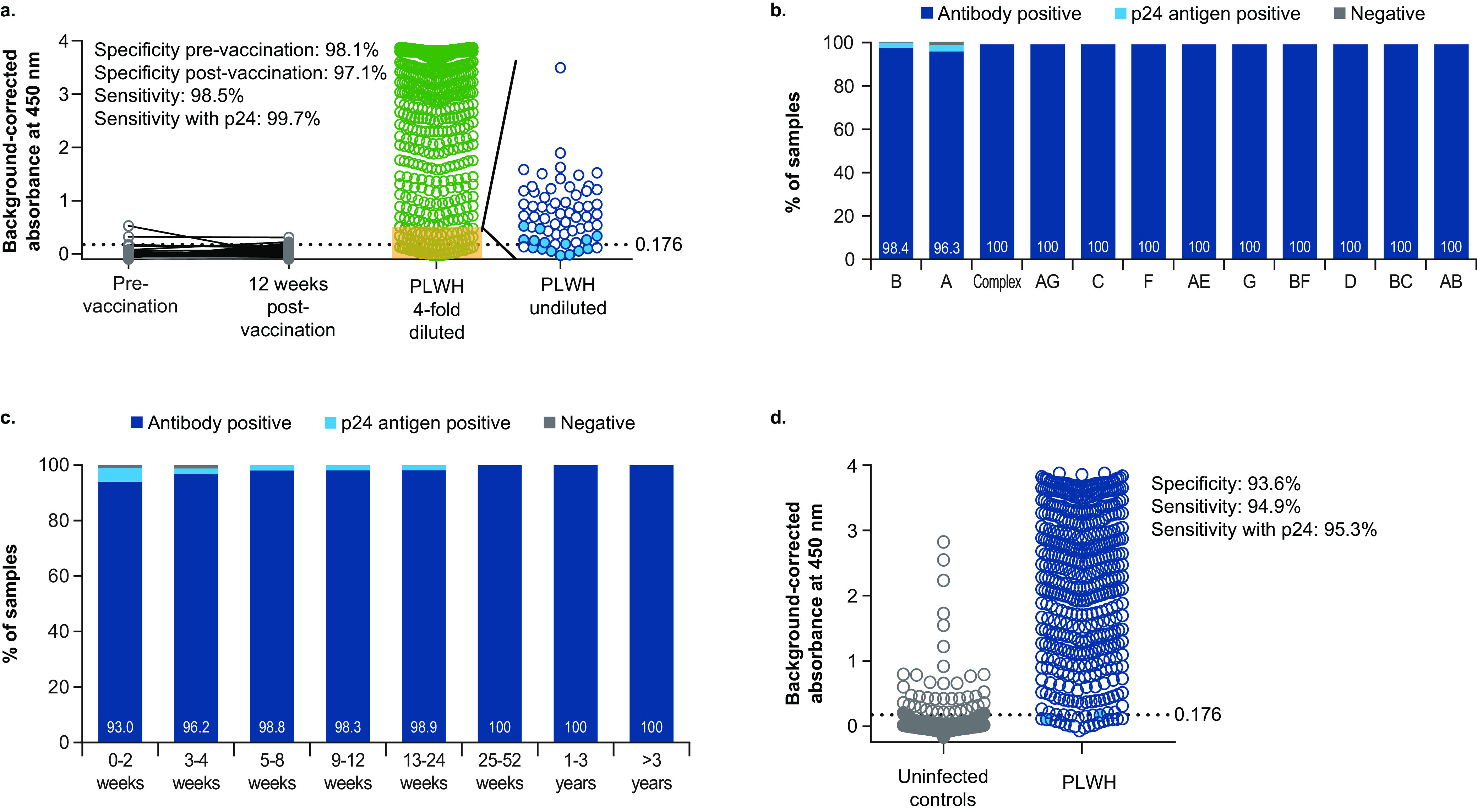
Performance evaluation of the DHIVAx assay. (a) Overall performance of the DHIVAx assay. Sensitivity of each polypeptide antigen to detect infection in treatment-naive individuals living with known HIV-1 infection (AMBER, *n* = 600). Samples were initially tested 4-fold diluted (green circles) and all samples with blank-corrected Abs_4__50nm_ were retested undiluted (blue circles). Samples with a positive p24 antigen detection test are indicated as closed blue circles. The specificity of each antigen was evaluated in individuals who participated in HIV vaccine studies (TRAVERSE and ASCENT, *n* = 109). Dotted horizontal line represents the threshold for positivity (0.176). (b) Sensitivity by HIV-1 clade. (c) Sensitivity by time since diagnosis. (d) Performance of the DHIVAx assay in the World Health Organization HIV specimen evaluation panel. Dotted horizontal line represents the threshold for positivity (0.176). PLWH, people living with HIV.

Performance of a DHIVAx assay, more specifically a double-antigen bridging (sandwich) ELISA combining the polypeptide antigens gp41e, p31, p17, and Nef, was assessed in 600 individuals with known HIV infection and resulted in a specificity of 98.1% (95% CI: 93.5% to 99.7%) in healthy controls (97.1% [95% CI: 91.8% to 99.2%] at 12 weeks after vaccination) and a sensitivity of 98.5% (95% CI: 97.2% to 99.2%; Fig. 2A). The sensitivity increased to 99.7% (95% CI: 98.8% to 99.9%) when p24 antigen detection was also included. The DHIVAx assay similarly recognized samples from seropositive individuals from different geographical origins and who were infected with different HIV-1 clades (Fig. 2B and Table S3). Among recently diagnosed individuals (≤12 weeks since the first diagnosis), 2 of 251 samples were negative for the DHIVAx assay when p24 antigen detection was included (Fig. 2C and Table S4); 8 of 251 samples were negative without p24 antigen detection. The sensitivity for the 349 samples from individuals with >12 weeks since the first diagnosis was 99.7% (100% when p24 antigen detection was included). While 91 of the 109 (83.5%) postvaccination samples from uninfected individuals were found positive in the Abbott ARCHITECT HIV Ag/Ab Combo assay (Fig. S1), no significant decrease in specificity postvaccination was observed for the DHIVAx assay, confirming that the assay was insensitive to vaccine-induced seroreactivity.

The DHIVAx assay was also used to test the World Health Organization (WHO) HIV specimen evaluation panel, which included samples from 736 uninfected individuals and 450 individuals with known HIV infection. In this panel, the specificity was 93.6% (95% CI: 91.6% to 95.3%) and the sensitivity was 94.9% (95% CI: 92.4% to 96.7%); when p24 antigen detection was also included, the sensitivity increased to 95.3% (95% CI: 93.0% to 97.1%; Fig. 2D).

### Longitudinal performance of the double-antigen bridging ELISA.

**FIG 3:**
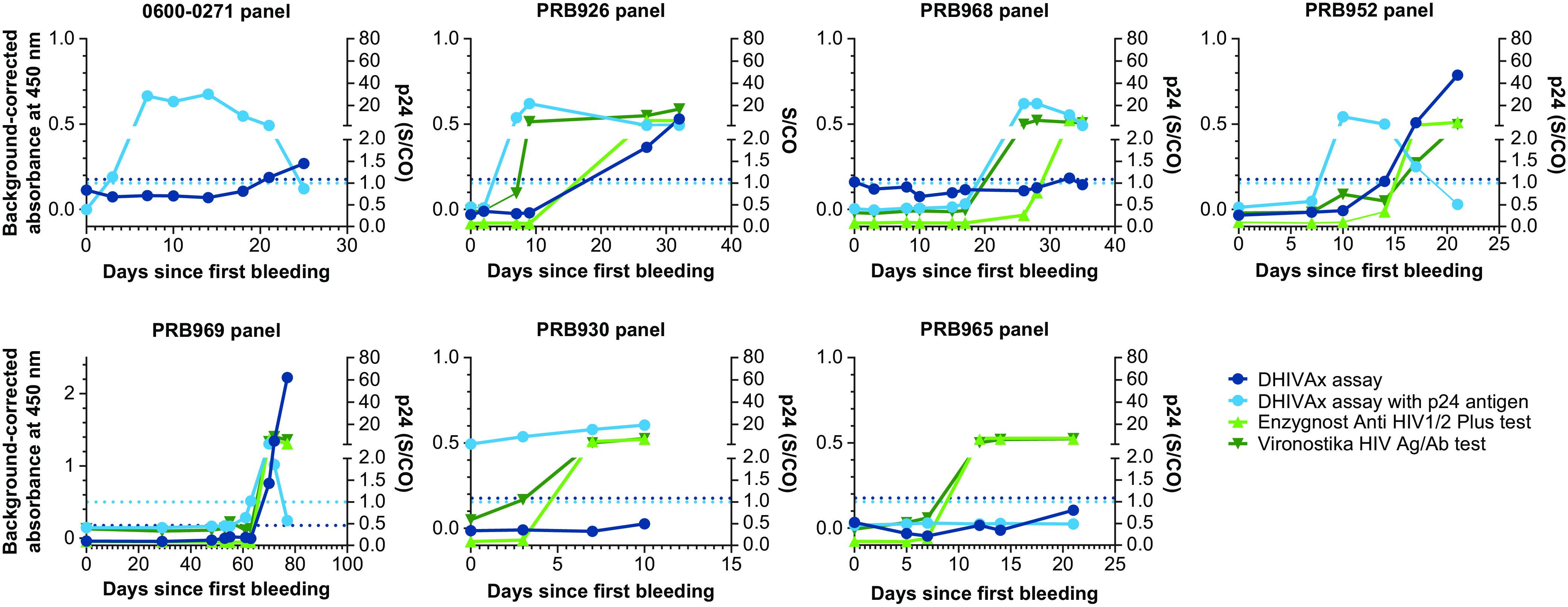
Performance of DHIVAx assay in seroconversion panels, including p24 antigen data and 2 reference tests. Dotted horizontal lines represent the thresholds for positivity for the DHIVAx assay (0.176; left *y* axis) and DHIVAx assay with p24 antigen detection (1.0; right *y* axis).

To determine how soon postinfection HIV-specific antibodies were detected with the DHIVAx assay, several well-characterized seroconversion panels were obtained from LGC SeraCare. As shown in Fig. 3, the DHIVAx assay reacted positively as soon as or before the p24 antigen became undetectable for the 0600-0271, PRB-926, PRB-968, and PRB-952 panels. For the PRB-969 and PRB-930 panels, the p24 antigen remained detectable on the last day of sampling, while the DHIVAx assay remained negative. For the PRB-965 panel, both the p24 antigen and the DHIVAx assay remained negative across all time points, while the Enzygnost Anti HIV1/2 Plus and Vironostika HIV Ag/Ab assays were positive on Days 12, 14, and 21.

**FIG 4:**
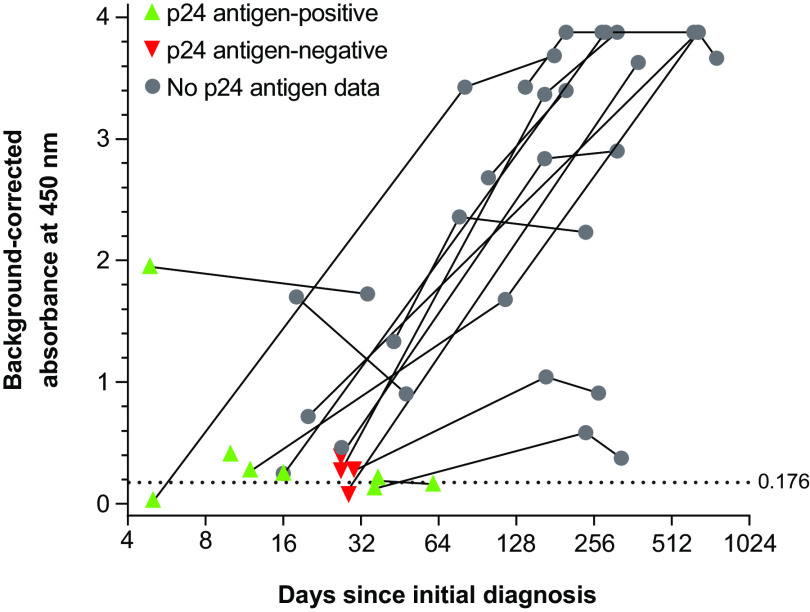
Assessment of DHIVAx assay in longitudinal follow-up samples collected from individuals with known HIV infection. Dotted horizontal line represents the threshold for positivity (0.176).

Assessment of the response dynamics in the DHIVAx assay was performed using a longitudinal sample series from 18 patients with documented seroconversion (Fig. 4). In most patients, the signal increased strongly over time and all samples, except 1, that were still negative with the DHIVAx assay were found to be positive for p24 antigen.

## DISCUSSION

In this study, we identified a number of polypeptide antigens derived from HIV-1 that could be used in diagnostic assays to accurately determine the presence of an HIV infection irrespective of the vaccination status of the tested individual. The antigens in the DHIVAx assay are known HIV-1 antigens, and all are known to be immunogenic to some extent (22); however, this is the first time they have been described in the context of VISR. The combination comprising polypeptide antigens derived from gp41e, p31, p17, and Nef showed a good sensitivity of 98.5% to the majority of HIV-1 clades and throughout all stages of infection and a specificity of 98.1% in uninfected healthy controls and 97.1% at 12 weeks after full vaccination. These observations are interesting for p31 and p17 because both proteins are encoded by the HIV vaccine used in this study and T-cell responses have been observed against these antigens; therefore, they were expected to be immunogenic and induce VISR. Together, these data suggest the combination of polypeptide antigens in the DHIVAx assay can distinguish between anti-HIV antibodies generated due to an HIV infection and anti-HIV antibodies induced following administration of an HIV vaccine (i.e., VISR) and show accuracy that approaches the accuracy of current fourth-generation HIV serological tests, which demonstrate sensitivity and specificity of >99% (23, 24). Therefore, this combination could be used in any population and could reveal HIV infection in vaccinated individuals without interference from VISR (i.e., can accurately detect breakthrough infections and minimize false positives). Ideally, it could be used in a high-prevalence setting with high vaccine uptake, as under these circumstances, VISR will have the greatest impact. In addition, since such a setting is more likely to be found in low- and middle-income countries, alternative solutions based on molecular testing algorithms are not feasible or affordable.

Current fourth- and fifth-generation HIV tests are designed to detect both p24 (which is detectable 2 to 3 weeks after HIV infection and then wanes after a month or 2 [25]) and the development of host antibodies against HIV. We observed in seroconversion panels and longitudinal samples that the response against the DHIVAx assay antigens steadily increased over time and, in most cases, the DHIVAx assay could detect antibodies to the polypeptide antigens before or as the p24 antigen became undetectable. Data from the seroconversion panels also demonstrated that, whereas Env-based serological assays overlap significantly with the p24 detection window, this is not the case for the DHIVAx assay, with both markers (antigen and antibodies) being continuously detectable in most cases. The addition of p24 detection to the DHIVAx assay in the next version of the assay will therefore be important to ensure early and continued detection of HIV infection.

Although a limitation of our work is that potential antigens for inclusion in the DHIVAx assay were evaluated against only the Janssen mosaic-based HIV-1 vaccine, it is likely that the combination of selected antigens included in DHIVAx will also work well in the context of other Env-based HIV vaccines. However, differences may exist for future HIV vaccine candidates. Furthermore, although HIV-1 RT was not retained as a suitable marker in our DHIVAx assay due to the presence of vaccine-induced antibodies against this antigen in individuals who received the mosaic-based vaccine regimen, it was a very strong marker for HIV infection. Therefore, RT could be one of the markers of choice if an HIV vaccine that does not contain pol-encoded proteins, in particular RT, is considered. This has been the case for several investigational HIV vaccines in the past (10, 26, 27) and for new mRNA-based HIV-1 vaccines in development (28).

The suboptimal specificity of Nef with indirect ELISA (96.3%) and the impact on the double-antigen bridging ELISA need additional investigation to determine whether this was caused by the binding of non-HIV antibodies to Nef itself or remaining impurities in the Nef protein product. The Nef protein used in the indirect ELISA was only 78.1% pure, and no purification steps were performed after affinity purification on the nickel Sepharose column. Data from the WHO HIV specimen evaluation panel confirmed there is a desire to further improve the specificity of the DHIVAx assay. As a first next step, we suggest optimizing the protein production and purification procedures, as this was not part of our evaluation and could significantly contribute to the overall performance of the assay.

Data from the WHO HIV specimen evaluation panel suggested that the sensitivity of the DHIVAx assay can benefit from further improvement. It is important to consider that, of the 23 false negatives, 10 individuals were treated with highly active antiretroviral therapy, some of them for multiple years. It is well known that the HIV antibody response declines during treatment and some patients even serorevert (~2% for those on ART for ≥9 years and 12% for those who started ART in the early phase of infection) (29, 30). While this might have a limited impact on Env-based serological tests, the effect might be more pronounced for the antigens proposed as part of DHIVAx, especially p31 (30–32).

In conclusion, we demonstrated that the DHIVAx assay, which includes a combination of polypeptide antigens (gp41e, p31, p17, and Nef), enables the detection of HIV infection with promising specificity and sensitivity, early after infection and without VISR induced by the mosaic-based HIV-1 vaccine regimen. These antigens can readily be transferred to established HIV testing platforms, such as lateral flow assays and electro-chemiluminescence immunoassays. Although further technical advancements will be desired, our research has provided the groundwork for the development of new HIV tests that are unaffected by VISR. Translating these results into a final diagnostic product that can be made broadly accessible globally will facilitate the parallel introduction of future HIV vaccines.

## MATERIALS AND METHODS

### Sources of study samples and ethical approval.

Serum samples used in this work were collected during the following clinical studies: AMBER (treatment-naive individuals with known HIV-1 infection collected in 2015 and 2016 in the United States, Canada, and Europe; ClinicalTrials.gov Identifier: NCT02431247) (21), TRAVERSE (individuals without HIV-1 infection, collected in 2017 and 2018 in the United States and Rwanda; NCT02788045) (19), and ASCENT (individuals without HIV-1 infection, collected in 2017 to 2019 in the United States, Kenya, and Rwanda; NCT02935686) (20). These studies were conducted in accordance with the principles of Good Clinical Practice and the Declaration of Helsinki. The protocol and amendments were reviewed and approved by an institutional review board or independent ethics committee. Written informed consent was obtained from all individuals prior to participation, and all samples were deidentified.

Additional serum samples from individuals with known HIV infection were obtained from the Mayo Clinic (Jacksonville, FL, USA). Written informed consent was obtained from all individuals prior to participation, and all samples were decoded and deidentified before they were provided for research purposes.

The WHO HIV specimen evaluation panel consisted of 1187 serum samples with known HIV status (451 positives for HIV-1 and 736 negatives) collected in Africa, Asia, Europe, and South America in the 1990s. The panel was characterized according to a standard combination of assays (i.e., a standardized testing algorithm), and these reference testing results were used to determine the true HIV status of each specimen for the purpose of this performance evaluation. In this evaluation, the ELISAs used in parallel were: Enzygnost Anti-HIV 1/2 (Siemens Healthcare Diagnostics, Marburg, Germany), Vironostika HIV Uni-Form II Plus O (bioMérieux, Boxtel, The Netherlands), Genscreen ULTRA HIV Ag-Ab (Bio-Rad, Hercules, CA, USA), and VIDAS HIV Duo Quick (bioMérieux). WHO has consented to the use of the HIV specimen evaluation panel for the purposes of this project. The protocol was considered exempt from review by WHO Ethics Review Committee (ERC) given that the specimens were recorded in such a manner as to be unidentifiable by the investigators (ERC exemption summary dated 10 August 2020; ERC.0003415). As the majority of these specimens were obtained from blood banks in the 1990s and anonymized before use, individual informed consent information was not available. The use of these archived specimens was in accordance with the Council for International Organizations of Medical Sciences’ international ethical guidance. Written informed consent was obtained from individuals contributing to specimens obtained in recent years.

HIV-1 seroconversion panels PRB-926, PRB-930, PRB-965, PRB-968, PRB-952, and 0600-0271 were purchased from LGC SeraCare (Gaithersburg, MD, USA).

A series of 43 sequential plasma samples from 18 patients with documented seroconversion were selected from the Biobank of the Aids Reference Laboratory of Ghent University. Samples were collected in Belgium between 2009 and 2014. This Biobank received approval from the ethical committee of Ghent University Hospital on February 6, 2019 (reference number 2018/1532). All patients provided written informed consent for scientific research (33).

### Study samples.

For antigen selection experiments, a set of 10 serum samples from individuals with known HIV infection were obtained from the Mayo Clinic. This was complemented with 10 samples collected prevaccination and 10 samples collected 12 weeks after the last vaccination from participants of the TRAVERSE and ASCENT HIV vaccine studies (19, 20). The vaccination regimen in these studies consisted of 2 immunizations with Ad26.Mos.HIV or Ad26.Mos4.HIV at Day 0 and Week 12, followed by 2 concomitant immunizations of adjuvanted clade C gp140 and Ad26.Mos.HIV or adjuvanted clade C gp140 + Mosaic gp140 and Ad26.Mos4.HIV at Weeks 24 and 48, respectively.

Performance evaluation of individual indirect ELISAs and the double-antigen bridging ELISA was performed on a set of serum samples from 600 individuals with known HIV-1 infection from the AMBER study (21). All study participants had a viral load >1,000 copies/mL, confirming their HIV-1 infection status, and were treatment naive. Additionally, serum samples were included from 109 healthy individuals without HIV-1 infection who participated in TRAVERSE and ASCENT HIV vaccine studies, with samples collected prevaccination and 12 weeks after the last vaccination (19, 20).

An additional WHO HIV specimen reference panel was included, consisting of 451 HIV-1–positive and 736 HIV-1–negative serum/plasma specimens of African, Asian, European, and Latin American origin. The panel was characterized by a standardized testing algorithm consisting of 2 ELISAs (Enzygnost Anti-HIV 1/2 and Vironostika HIV Uni-Form II Plus O or Genscreen ULTRA HIV Ag-Ab and VIDAS HIV Duo Quick) followed by INNO-LIA HIV I/II Score (Fujirebio, Zwijnaarde, Belgium) confirmation for specimens with either dually reactive results on both ELISAs or with discrepant ELISA results. Specimens that were negative by the line immunoassay were further tested on INNOTEST HIV Antigen MAb (Fujirebio) ELISA. These reference testing results were utilized to determine the true HIV status of each specimen for the purpose of this performance evaluation.

HIV-1 seroconversion panels contained plasma samples collected serially early after HIV-1 infection. At each blood collection, HIV RNA, p24, and antibodies were assessed by commercial diagnostic kits by the respective manufacturers, and this information was provided with the panels. Additionally, the INNOTEST HIV Antigen MAb (Fujirebio), Enzygnost Anti-HIV 1/2 Plus (Siemens Healthcare Diagnostics), and Vironostika HIV Ag/Ab (bioMérieux) tests were performed on all samples.

The longitudinal sample series comprised 43 plasma samples from 18 patients with documented seroconversion. One to 3 samples were tested per patient, and the mean total follow-up period was 263 days (range: 10–766). The first sample collected from each patient (Day 0) showed a reactive p24 antigen test but no detectable antibodies in the INNO-LIA HIV I/II Score (33).

All samples were shipped on dry ice (temperature between −78°C and −110°C) and stored at −80°C until analysis.

### Recombinant proteins.

All recombinant proteins were produced in an E. coli expression system by Kaneka Eurogentec (Seraing, Belgium). A His_6_ tag was included in the proteins for purification on a Ni^++^ Sepharose column. Biotinylation of these recombinant proteins was also performed by Kaneka Eurogentec using the water-soluble EZ-Link Sulfo-NHS-LC-Biotin (Thermo Scientific, Waltham, MA, USA). Protein sequences are listed in Table S5.

### Indirect ELISA.

IgG antibody levels against the different antigens were assessed using indirect ELISAs. These ELISAs were performed using flat-bottomed polystyrene plates (Maxisorp Immuno Plate, Nunc, Denmark) coated with the individual proteins, diluted to the concentration indicated in Table 1, and incubated overnight at 4°C. For every sample, a condition with no coated protein was included to assess the sample-specific background signal. After washing with PBS with 0.05% Tween 20 (PBS-T), the plate was blocked with block buffer (Blocker Casein in PBS; Thermo Fisher Scientific, Breda, the Netherlands) for 1 h at room temperature. Serum samples, diluted 100-fold in sample diluent (block buffer + 5% Triton X-100), were added in duplicate and incubated for 1 h at room temperature. Plates were washed 5 times with PBS-T, after which the secondary antibody solution was added to each well. The solution contained an affinity-purified donkey anti-human IgG (H+L) peroxidase conjugate (Jackson Immuno Research Europe Ltd., Newmarket, UK) diluted 1:10,000 in block buffer. The reaction mixture was incubated at room temperature for 30 min. At the end of the incubation period, the plates were rinsed 5 times with washing buffer and treated with 100 μL 1-Step Ultra TMB-ELISA Substrate Solution (Thermo Fisher Scientific). After 10 min of incubation, the colorimetric reaction was stopped with 100 μL 1N HCl. The plate was then read by a microplate reader at a wavelength of 450 nm. For every sample and antigen, the final value was calculated by subtracting the sample-specific background from the measured absorbance. For each antigen, an antigen-specific threshold for positivity was calculated based on Receiver Operating Characteristic analysis by selecting the value with the maximal Youden index (34).

**TABLE 1:** Proteins used in the indirect ELISAs^*a*^

Antigen name	Commercial supplier	Supplier ID	Coating concn	Coating buffer
Proteins used for antigen selection				
Gag-p55	Abcam	ab63995	2.5 μg/mL	PBS
Gag-p17	Abcam	ab63981	1 μg/mL	PBS
Gag-p15	Abcam	ab63994	1 μg/mL	PBS
RT	Abcam	ab63979	1 unit/mL	PBS
p31 integrase	Abcam	ab173265	2.5 μg/mL	PBS
Protease	Abcam	ab84117	1 μg/mL	PBS
Nef	Abcam	ab63996	1 μg/mL	PBS
His_6_-gp41e	NA	NA	0.1 μg/mL	0.1 M carbonate
Proteins used for performance evaluation of selected antigens				
Gag-p17	RPC	AHIV 205	0.5 μg/mL	PBS
p31 integrase	Abcam	ab173265	0.5 μg/mL	PBS
His_6_-Nef	In-house	In-house	1 μg/mL	PBS
His_6_-gp41e	In-house	In-house	0.1 μg/mL	0.1 M carbonate

a gp41e, glycoprotein 41 endodomain; NA, not applicable; RPC, RPC Diagnostic Systems, Ltd. (Nizhny Novgorod, Russia); RT, reverse transcriptase.

### Combination double-antigen bridging ELISA.

Bridging ELISAs were performed using flat-bottomed polystyrene plates coated with a mixture of proteins, diluted to the concentration indicated in Table 2 in 0.1 M carbonate buffer, and incubated overnight at 4°C. After washing with PBS-T, plates were blocked with block buffer for 1 h at room temperature. 20 μL of serum samples and 80 μL of sample diluent (block buffer + 0.5% Triton X-100) were added in duplicate and incubated for 30 min at room temperature. On every plate, a blank (100 μL of sample diluent) was included. Subsequently, 50 μL of the biotinylated detection proteins, diluted to the concentration indicated in Table 2 in block buffer, were added, and the plates were further incubated for 30 min at room temperature. Plates were washed 3 times with PBS-T, and the streptavidin-HRP solution was added to each well. The solution contained a peroxidase-conjugated streptavidin (Jackson Immuno Research Europe Ltd.) diluted 1:20,000 in block buffer. The reaction mixture was incubated at room temperature for 30 min. At the end of the incubation period, the plates were rinsed 3 times with washing buffer and treated with 100 μL 1-Step Ultra TMB-ELISA Substrate Solution. After 10 min of incubation, the colorimetric reaction was stopped with 100 μL 1N HCl. The plate was read by a microplate reader at a wavelength of 450 nm. For every sample, the final value was calculated by subtracting the plate blank from the measured absorbance. The threshold for positivity was calculated based on Receiver Operating Characteristic analysis, by selecting the value with the maximal Youden index (34).

**TABLE 2:** Proteins used in the combo double-antigen bridging ELISA^*a*^

Antigen name	Commercial supplier	Supplier ID	Coating concn	Detection concentration^*b*^
His_6_-p17	In-house	In-house	5 μg/mL	NA
His_6_-p31	In-house	In-house	5 μg/mL	NA
His_6_-Nef	In-house	In-house	0.5 μg/mL	NA
MBP-gp41e	In-house	In-house	2 μg/mL	NA
Biotinylated His_6_-p17	In-house	In-house	NA	1 μg/mL
Biotinylated p31	RPC	AHIV-b-108	NA	1 μg/mL
Biotinylated His_6_-Nef	In-house	In-house	NA	1 μg/mL
Biotinylated MBP-gp41e	In-house	In-house	NA	1 μg/mL

a gp41e, glycoprotein 41 endodomain; NA, not applicable; RPC, RPC Diagnostic Systems, Ltd. (Nizhny Novgorod, Russia).

b Final concentration after addition to the sample preincubated in the well.

### p24 antigen assay and INNO-LIA HIV I/II.

The presence of the p24 HIV core antigen was determined using the INNOTEST HIV Antigen MAb (Fujirebio). The INNO-LIA HIV I/II Score assay (Fujirebio) allowed differentiation and comparison in antibody reactivity to individual HIV antigens. Both assays were performed according to the manufacturers’ instructions.

### Data availability.

All data generated or analyzed during this study are included in this published article and its supplementary information files.
