# Supplementary material for: Towards Novel HIV-1 Serodiagnostic Tests without Vaccine-Induced Seroreactivity
Source: Microbiol Spectr. 2023 May 24;11(3):e00715-23. doi: 10.1128/spectrum.00715-23 (PMC10269835; doi:10.1128/spectrum.00715-23)
Supplement: Supplemental file 1 — Tables S1 to S5 and Fig. S1. Download spectrum.00715-23-s0001.docx, DOCX file, 0.07 MB [file spectrum.00715-23-s0001.docx]

**Towards novel HIV-1 serodiagnostic tests without vaccine-induced seroreactivity**

Ole Lagatie, Dax Lauwers, Harvinder Singh, Fien Vanroye, Daniel J. Stieh, Johan Vingerhoets, Ludo Lavreys, Valérie Oriol-Mathieu, Will Colón, Chris Verhofstede, Koen Vercauteren, Dorien Van den Bossche, Maria Grazia Pau

**Supplementary Material**

**Table S1.** Combined sensitivity for gp41e, p31, p17, and Nef by HIV-1 clade.

| **HIV-1 clade** | **n** | **Sensitivity (%)** |
| --- | --- | --- |
| B | 432 | 98.4% |
| A | 54 | 96.3% |
| COMPLEX | 33 | 100.0% |
| AG | 32 | 100.0% |
| C | 15 | 100.0% |
| F | 10 | 100.0% |
| AE | 9 | 100.0% |
| G | 5 | 100.0% |
| BF | 5 | 100.0% |
| D | 2 | 100.0% |
| BC | 2 | 100.0% |
| AB | 1 | 100.0% |

gp41e, His_6_-gp41 endodomain.

**Table S2.** Combined sensitivity for gp41e, p31, p17, and Nef by time since diagnosis.

| **Time since diagnosis** | **n** | **Sensitivity (%)** | | **Sensitivity (%) including**  **p24 antigen** | |
| --- | --- | --- | --- | --- | --- |
| 0-2 weeks | 56 | 96.4% | 96.4% | 98.2% | 97.6% |
| 3-4 weeks | 53 | 92.5% |  | 96.2% |  |
| 5-8 weeks | 82 | 97.6% |  | 97.6% |  |
| 9-12 weeks | 60 | 98.3% |  | 98.3% |  |
| 13-24 weeks | 88 | 100.0% | 100.0% | 100.0% | 100.0% |
| 25-52 weeks | 81 | 100.0% |  | 100.0% |  |
| 1-3 years | 76 | 100.0% |  | 100.0% |  |
| >3 years | 104 | 100.0% |  | 100.0% |  |
| Total | 600 | 98.5% | 98.5% | 99.0% | 99.0% |

gp41e, His_6_-gp41 endodomain.

**Table S3.** Sensitivity of the DHIVAx assay by HIV-1 clade.

| **HIV-1 clade** | **n** | **Sensitivity (%)** |
| --- | --- | --- |
| B | 432 | 98.4% |
| A | 54 | 96.3% |
| COMPLEX | 33 | 100.0% |
| AG | 32 | 100.0% |
| C | 15 | 100.0% |
| F | 10 | 100.0% |
| AE | 9 | 100.0% |
| G | 5 | 100.0% |
| BF | 5 | 100.0% |
| D | 2 | 100.0% |
| BC | 2 | 100.0% |
| AB | 1 | 100.0% |

Assessed using a double-antigen bridging ELISA.

**Table S4.** Sensitivity of the DHIVAx assay by time since diagnosis.

| **Time since diagnosis** | **n** | **Sensitivity (%)** | | **Sensitivity (%) including p24 antigen** | |
| --- | --- | --- | --- | --- | --- |
| 0-2 weeks | 56 | 92.9% | 96.8% | 98.2% | 99.2% |
| 3-4 weeks | 53 | 96.2% |  | 98.1% |  |
| 5-8 weeks | 82 | 98.8% |  | 100.0% |  |
| 9-12 weeks | 60 | 98.3% |  | 100.0% |  |
| 13-24 weeks | 88 | 98.9% | 99.7% | 100.0% | 100.0% |
| 25-52 weeks | 81 | 100.0% |  | 100.0% |  |
| 1-3 years | 76 | 100.0% |  | 100.0% |  |
| >3 years | 104 | 100.0% |  | 100.0% |  |
| Total | 600 | 98.5% | 98.5% | 99.7% | 99.7% |

Assessed using a double-antigen bridging ELISA.

**Table S5.** Sequences of recombinantly produced proteins.

| **Protein** | **Sequence** |
| --- | --- |
| His_6_-gp41 endodomain | MAHHHHHH ENLYFQG NRVRQGYSPL SFQTHLPTPR GPDRPEGIEE EGGERDRDRS IRLVNGSLAL IWDDLRSLCL FSYHRLRDLL LIVTRIVELL GRRGWEALKY WWNLLQYWSQ ELKNSAVSLL NATAIAVAEG TDRVIEVVQG ACRAIRHIPR RIRQGLERIL L |
| His_6_-p17 | MAHHHHHHGS DDDDK GARASVLSGG ELDRWEKIRL RPGGKKKYKL KHIVWASREL ERFAVNPGLL ETSEGCRQIL  GQLQPSLQTG SEELRSLYNT VATLYCVHQR IEIKDTKEAL DKIEEEQNKS KKKAQQAAAD TGHSNQVSQNY |
| His_6_-p31 | MAHHHHHHGS DDDDK FLDGIDKAQD EHEKYHSNWR AMASDFNLPP VVAKEIVASC DKCQLKGEAM HGQVDCSPGI WQLDCTHLEG KVILVAVHVA SGYIEAEVIP AETGQETAYF LLKLAGRWPV KTIHTDNGSN FTGATVRAAC WWAGIKQEFG IPYNPQSQGV VESMNKELKK IIGQVRDQAE HLKTAVQMAV  FIHNFKRKGG IGGYSAGERI VDIIATDIQT KELQKQITKI QNFRVYYRDS RNPLWKGPAK LLWKGEGAVV IQDNSDIKVV PRRKAKIIRD YGKQMAGDDC VASRQDED |
| His_6_-Nef | MAHHHHHHGS DDDDK MGGKWSKSSV VGWPTVRERM RRAEPAADGV GAASRDLEKH GAITSSNTAA TNAACAWLEA QEEEEVGFPV TPQVPLRPMT YKAAVDLSHF LKEKGGLEGL IHSQRRQDIL DLWIYHTQGY FPDWQNYTPG PGVRYPLTFG WCYKLVPVEP DKVEEANKGE NTSLLHPVSL HGMDDPEREV LEWRFDSRLA FHHVARELHP EYFKNC |
| MBP-gp41 endodomain | MKIEEGKLVI WINGDKGYNG LAEVGKKFEK DTGIKVTVEH PDKLEEKFPQ VAATGDGPDI IFWAHDRFGG YAQSGLLAEI TPDKAFQDKL YPFTWDAVRY NGKLIAYPIA VEALSLIYNK DLLPNPPKTW EEIPALDKEL KAKGKSALMF NLQEPYFTWP LIAADGGYAF KYENGKYDIK DVGVDNAGAK AGLTFLVDLI KNKHMNADTD YSIAEAAFNK GETAMTINGP WAWSNIDTSK VNYGVTVLPT FKGQPSKPFV GVLSAGINAA SPNKELAKEF LENYLLTDEG LEAVNKDKPL GAVALKSYEE ELAKDPRIAA TMENAQKGEI MPNIPQMSAF WYAVRTAVIN AASGRQTVDE ALKDAQTNSS SNNNNNNNNN NLG ENLYFQS NRVRQGYSPL SFQTHLPTPR GPDRPEGIEE EGGERDRDRS IRLVNGSLAL IWDDLRSLCL FSYHRLRDLL LIVTRIVELL GRRGWEALKY WWNLLQYWSQ ELKNSAVSLL NATAIAVAEG TDRVIEVVQG ACRAIRHIPR RIRQGLERIL L |

The His6-tag is indicated in green, the MBP tag in blue, the cleavage site for TEV protease in red, and the enterokinase cleavage site in orange. Protein sequences were based on NCBI Reference Sequences for HIV-1: NP_579895.1 for gp41, NP_579876.2 for p17, NP_705928.1 for p31, and P03406.3 for Nef. The MBP sequence was based on pMAL vectors (New England Biolabs, Ipswich, MA, USA).

MBP, maltose-binding protein; NCBI, National Center for Biotechnology Information.

**Figure S1.** A comparative example of performance assessment using the Abbott ARCHITECT^®^ HIV Ag/Ab Combo assay. At 12 weeks after vaccination, 90 of the 109 (83.5%) post-vaccination samples from uninfected individuals were found positive with this assay. Dotted horizontal line represents the threshold for positivity.
